# Trade-Offs between Stability and Activity of Glycosylated and Non-Glycosylated Polyester Hydrolases PHL7 and PHL7mut3

**DOI:** 10.1021/acsestengg.5c00272

**Published:** 2025-08-07

**Authors:** Lisa Fohler, Felix Faschingeder, Lukas Leibetseder, Ziyue Zhao, Abibe Useini, Norbert Sträter, Christian Sonnendecker, Tom A. Ewing, Antoine P. H. A. Moers, Marc W. T. Werten, Daan M. van Vliet, Mattijs K. Julsing, Wolfgang Zimmermann, Gerald Striedner

**Affiliations:** † Department of Biotechnology, Institute of Bioprocess Science and Engineering, 27270 BOKU University, Vienna 1190, Austria; ‡ Institute of Analytical Chemistry, 9180 Leipzig University, Leipzig 04103, Germany; § Institute of Bioanalytical Chemistry, Center for Biotechnology and Biomedicine, Leipzig University, Leipzig 04103, Germany; ∥ Wageningen Food & Biobased Research, 4508 Wageningen University & Research, Wageningen 6708, The Netherlands

**Keywords:** *Escherichia coli*, *Komagataella phaffii*, polyester hydrolases, PHL7mut3, PES-H1, enzymatic plastic recycling, *Pichia pastoris*

## Abstract

Plastic pollution has become a global environmental challenge, driving interest in enzymatic polyethylene terephthalate (PET) recycling by using polyester hydrolases. In this study, we produced the PET-degrading enzyme PHL7 and its variant PHL7mut3 in *Escherichia coli* and *Pichia pastoris* (syn. *Komagataella phaffii*) to investigate the impact of N-glycosylation on enzyme properties. While glycosylation upon expression in *P. pastoris* enhanced thermal stability, it reduced the catalytic activity of the enzymes, revealing a trade-off that adds complexity to the selection of the best-suited expression system. Additionally, we engineered *P. pastoris* to produce non-glycosylated enzyme variants by substituting the asparagine residues (N) at all three putative N-glycosylation sites with glutamine residues (Q). The non-glycosylated *P. pastoris*-produced enzymes showed a lower activity compared to those produced in *E. coli*, likely due to the differences in the amino acid sequence. The effects of glycosylation were less pronounced in PHL7mut3 than in PHL7, yet N-glycosylation strongly influenced the performance of both enzymes. We further demonstrate that the PET degradation performance of PHL7mut3 is less dependent on the buffer ionic strength than that of PHL7. The study emphasizes the need for the informed selection of a suitable expression host for polyester hydrolases to balance enzyme activity, thermostability, and production titer for applications in PET recycling.

## Graphical abstract

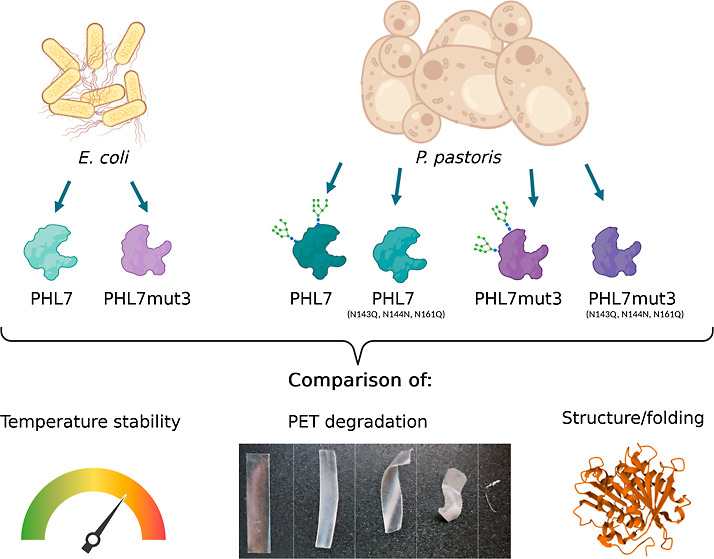

## Introduction

With approximately 4.9 billion tons of plastics in landfills and an additional 180 million tons added annually, the plastic waste problem is more pressing than ever.^1–3^ Advancing toward a truly circular economy is crucial, and for polyethylene terephthalate (PET), enzymatic recycling offers new possibilities for sustainable plastic waste management.^4^ The first PET hydrolase was described by Müller et al.^5^ in 2005. Now, 20 years later, a multitude of polyester-degrading enzymes, originating from various organisms, have been described.^6^

Metagenome-derived polyester hydrolases, such as LCC^7^ and PHL7,^8^ have garnered significant attention due to their high activity at elevated temperatures of up to 70 °C. Their engineering led to the development of optimized variants, including LCC^ICCG^,^9^ LCC-A2,^10^ Turbo-PETase,^11^ and PHL7mut3.^12,13^ PHL7mut3 has been designed to improve the activity and thermostability of PHL7 by the Q175E, L210T, and D233 K mutations. The improved stability and activity of these engineered hydrolases bring their industrial application for polyester recycling closer to reality.

Understanding the biochemical properties and influence of modifications on these enzymes is crucial for their successful production and deployment in industrial processes. A study by Shirke et al.^14^ investigated the influence of glycosylation on LCC, which revealed a positive impact on catalytic efficiency in PET hydrolysis by increasing stability at elevated temperatures and substrate concentrations. A similar effect for PHL7 and its mutants has not yet been reported. Since PHL7 and LCC differ notably in several properties—including a relatively low amino acid sequence identity (53%) and a marked difference in isoelectric point (PHL7 pI 5.5 vs LCC pI 9.2)—it is conceivable that other characteristics, like the expression behavior and the effect of glycosylation on the enzyme, differ as well. In the process of selecting an expression host for enzyme production, the effects of possible glycosylation by the host strain must be considered.

Two commonly used systems for recombinant protein production are based on *Escherichia coli* and *Pichia pastoris*. *E. coli* offers advantages such as rapid growth, inexpensive cultivation media, ease of genetic manipulation, and extensive knowledge about the organism. However, potential challenges include the lack of post-translational modifications (PTMs) and secretion, the formation of stable disulfide bonds only in the periplasm, and the production of endotoxins.^15^ For polyester-degrading enzymes, endotoxins are not a concern because of their nonpharmaceutical application. Although not actively secreted, these enzymes are released into the *E. coli* culture supernatant via cell lysis due to their cell-toxic nature.^16^ *P. pastoris* grows more slowly, resulting in longer production times, and genetic manipulation is slightly more complex. However, this yeast is capable of growing to very high cell densities, facilitating disulfide bond formation, efficiently secreting products into the fermentation supernatant and performing PTMs such as N- and O-glycosylation. Although glycosylation often enhances enzyme thermal stability, it can sometimes also result in reduced enzymatic activity.^17–19^

We compared the performances of PHL7 and PHL7mut3 produced in *E. coli* and *P. pastoris*. We anticipated that the yeast expression system may introduce N-glycosylations at the Asn-Xxx-Ser/Thr motifs (where Xxx is not Pro) within the enzyme sequence,^20^ potentially altering enzyme characteristics. We engineered variants of both enzymes lacking all three potential N-glycosylation sites to evaluate if those polyester hydrolases could be produced in *P. pastoris* with properties identical or close to those of the *E. coli*-produced enzymes. The results of this study provide insight into the influence of glycosylation on the catalytic performance of polyester hydrolases PHL7 and PHL7mut3 and the effect of the exchange of amino acids at the N-glycosylation sites.

## Materials and Methods

### Strains and Cloning

For the *E. coli*-produced enzymes E_PHL7 and E_PHL7mut3, the respective gene sequences, including a hexa-histidine tag (His-tag), were cloned into a pET30a.cer plasmid and introduced into the growth-decoupled *E. coli* strain e^x^-press V2 (enGenes Biotech, Vienna, Austria) via electroporation.

Corresponding codon-optimized genes for expression in *P. pastoris*, P_PHL7 and P_PHL7mut3, were synthesized by Genscript Biotech. Variants P_PHL7_ng and P_PHL7mut3_ng were generated by replacing asparagine (N) with glutamine (Q) at all three Asn-Xxx-Ser/Thr motifs. The four genes were cloned into respective pPIC9 plasmids (Invitrogen; Waltham, MA, US) via *Xho*I and *Eco*RI. The α-mating factor signal provided in this vector enabled the secretion of the protein into the medium. The expression vectors were linearized with *Sal*I and used to transform *P. pastoris* strain GS115 (Invitrogen, Waltham, MA, USA) via electroporation. Using colony PCR, Mut^+^ transformants were selected that showed correct integration at the *his4* locus.

All amino-acid sequences are shown in Supporting Information, S1.

### Expression in *E. coli*

The expression of enzymes in *E. coli* was carried out using the growth-decoupled enGenes e^x^-press V2 strain as described by Fohler et al.^13^ In short, the cultivation was performed in DASGIP benchtop reactors (Eppendorf SE, Hamburg, Germany), with the temperature set to 37 (±0.2) °C and pH control set to 7.0 (±0.1) and regulated by dosing 12.5% ammonia solution. The dissolved oxygen (DO) cascade was designed to maintain the DO at 30%. 500 mL of batch medium was inoculated with a cell suspension from a preculture equivalent to 25 optical density units. At the batch end, an exponential feed with a growth rate of 0.135 h^–1^ was initiated. At a cell dry weight of 40 g L^–1^ (∼OD 140), the feed profile was switched to a fixed feed rate of 0.08 g glucose min^–1^, and the protein production and growth decoupling (by expression of the T7 phage protein Gp2) were induced by adding 0.035 mM IPTG and 10 mM arabinose to the reactor, respectively. The cell suspension was harvested after 10 h of induction, and the cells were separated from the supernatant by centrifugation (18,000*g*, 30 min, 4 °C).

### Expression in *P. pastoris*

Fermentations with the *P. pastoris* mutant strains were performed in 2.5 L Bioflo 3000 fermenters (New Brunswick Scientific, New Jersey, US). With the PHL7mut3 strain, one larger fermentation was conducted in 5.5 L of Bioflo 310 (New Brunswick Scientific, New Jersey, US). The strains were grown in a minimal salts medium^21^ according to methods described previously.^22^ The pH of all fermentations was maintained at 5.0 during the glycerol batch phase and was increased to 5.5 h half an hour prior to methanol induction, except for the last PHL7mut3_ng fermentation, where 30 min after methanol induction the pH was increased to 5.5. The glycerol batch phase lasted approximately 24 h, and the methanol fed-batch phase lasted 92 to 94 h. The cells were separated from the broth by centrifugation for 60 min at 16,900 *g*, followed by microfiltration of the supernatant over a 0.2 μm pore size filter. Wet cell weights were in the range of 220 g L^–1^ for PHL7mut3 and 290 g L^–1^ for PHL7mut3_ng.

### Enzyme Purification

For purification, the sterile filtered fermentation supernatant was used. For P_PHL7mut3 and P_PHL7mut3_ng, the pH of the supernatants was adjusted to 8 and centrifuged for 10 min at 10,000*g* and 4 °C prior to further purification steps. First, a Ni-NTA chromatography with a 5 mL HisTrap FF column (Cytiva, Marlborough MA, USA) with a flow rate of 5 mL min^–1^ was performed. The binding buffer consisted of 50 mM sodium phosphate and 200 mM NaCl, pH 7.4 (buffer A), and for the elution buffer (buffer B), 250 mM imidazole was added to the binding buffer. The column was equilibrated with 5 column volumes (CV) of 8% B prior to sample injection (50 mL). The sterile filtered supernatants were loaded onto a ÄKTA purifier system (Cytiva, Marlborough, MA, USA). The column was washed with 5 CV 8% B, and a step elution with 5 CV 16% B and 7 CV 100% B was performed. The fraction containing the recombinant protein was collected and concentrated with an Amicon 10 kDa cutoff ultrafiltration filter (Merck KGaA, Darmstadt, Germany). To remove imidazole and residual contaminants, size exclusion chromatography with a Hiload 26/60 Superdex 200 pg column (Cytiva, Marlborough, MA, USA) with buffer A was performed. All purified proteins were stored at 4 °C.

### SDS-PAGE and Determination of Protein Concentration

To confirm the successful purification, all samples were analyzed via sodium dodecyl sulfate-polyacrylamide gel electrophoresis (SDS-PAGE) and stained with Coomassie Brilliant Blue R250, as described by Stargardt et al.^23^ For the cleavage of N-glycans, the samples were treated with PNGase F (New England Biolabs, NEB, Ipswitch, MA, USA) according to the manufacturer’s protocol. The cleavage was carried out with both natively folded proteins and proteins that were denatured prior to PNGase F treatment. In short, for the denaturation, 9 μL of glycoprotein solution (15 μg protein) was mixed with 1 μL of glycoprotein denaturing buffer (10×, New England Biolabs, NEB, Ipswitch, MA, USA) and incubated at 100 °C for 10 min. Subsequently, the mixture was chilled, centrifuged, and mixed with 2 μL of GlycoBuffer 2 (10×, New England Biolabs, NEB, Ipswitch, MA, USA), 2 μL of 10% NP-40, and 2 μL of dH2O. For the cleavage of glycans, 1 μL of PNGase F was added, and the reaction mixtures were incubated at 37 °C for 1 h. For the cleavage of glycans on natively folded proteins, 18 μL of glycoprotein solution (15 μg protein) was mixed with 2 μL of GlycoBuffer 2 (10×) and 3 μL of PNGase F directly and incubated at 37 °C for 24 h. The protein concentration of the purified enzymes was determined by the Bradford method using the ROTIQuant assay kit (Carl Roth GmbH+ Co. KG, Karlsruhe, Germany) according to the enclosed instructions. The photometric analysis of the assays was carried out with a Synergy MX plate reader and Gen5 control software (BioTek Instruments, Inc., Winooski, VT, USA) at 450 and 590 nm.

### Protein Melting Temperature Determination

For the determination of the protein melting temperature (*T*_m_), nano-differential scanning fluorometry analysis was carried out using the Prometheus NT.48 (NanoTemper Technologies, Munich, Germany). The measurement was performed in 50 mM sodium phosphate and 200 mM sodium chloride (pH 7.4) using a protein concentration of 0.15 mg mL^–1^. A heating ramp from 20 to 95 °C with a slope of 1 °C min^–1^ was used. The instrument has a fixed excitation wavelength of 285 nm in combination with emission wavelengths of 330 and 350 nm.

### Protein and N-Glycosylation Structure Prediction

The protein folding of the different enzyme variants without glycan molecules was predicted using AlphaFold3, and the resulting structural information was visualized with the PyMOL software.^24^ For the display of the possible conformations of the glycan structures, the complete conformational library for Man_9_GlcNAc_2_ ^25^­(for the structure see, Supporting Information, S3) in conjugation with the enzyme was modeled using the computational method described by Turupcu and Oostenbrink.^26^ As Man_9_GlcNAc_2_ is a well-defined structure with available parametrization for molecular dynamics simulations, it was used here to gain initial insights into possible effects of N-glycosylation on enzyme properties before the start of cloning and expression.

### Crystal Structure

The samples E_PHL7mut3, P_PHL7mut3, and P_PHL7mut3_ng were concentrated to 8 mg mL^–1^ in 50 mM phosphate buffer (pH 7.4) containing 200 mM NaCl, prior to crystallization. A crystallization screen was conducted using approximately 400 buffer conditions in 96-well plates. The setups involved mixing equal volumes of protein and reservoir solution in a sitting-drop vapor diffusion approach, equilibrated against 100 μL of reservoir solution. Crystallization drops (200 nL final volume) were prepared using a Mosquito Xtal3 pipetting robot (SPT Labtech, Melbourn, England). Plates were stored at 19 °C and regularly monitored for crystal growth. Conditions yielding optimal crystals were manually reproduced using a hanging-drop vapor diffusion method at the microliter scale. Crystallization drops (2 μL of protein and 1 μL of reservoir solution) were equilibrated against 500 μL of reservoir solution. The best crystals of E_PHL7mut3 were obtained in 0.2 M NaCl, 0.1 M HEPES, pH 7.5, 25% w v^–1^ polyethylene glycol (PEG) 3350, P_PHL7mut3 in 0.2 M Li_2_SO_4_, 0.1 M Tris–HCl (pH 8.5), and 30% w v^–1^ PETPEG 4000, and P_PHL7mut3_ng in 0.1 M Bicine (pH 9.0) and 5% w v^–1^ PEG 6000 crystallization conditions.

Crystals appeared within 3–5 days at 19 °C. Single crystals, measuring 0.5 and 1 mm, were flash-frozen in liquid nitrogen. For cryoprotection, 15% PEG 400 was used exclusively for E_PHL7mut3, while no cryoprotectant was required for P_PHL7mut3 or P_PHL7mut3_ng.

Diffraction data were collected at beamlines P13 and P14 at PETRA III (DESY synchrotron, Hamburg, Germany). Data processing, including indexing, integration, and scaling, was performed using XDS (version 10 January 2022)^27^ and STARANISO (version 2.3.74) as implemented in ISPyB^28^ at DESY. Molecular replacement was employed to place the chains of the three structures, using the known structure PDB 7NEI as the search model, executed with Phaser within CCP4i2 (CCP4 version 8.0).^29^ Refinement was conducted using jelly body refinement in REFMAC (version 5.8.0425).^30^ Model building was completed in Coot (version 0.9.8.93),^31^ and final refinements were carried out in Phenix (version 1.20.1_4487).^32^ Data collection and refinement statistics are listed in Table S4.

### Small-Scale Amorphous PET Film Degradation Assays

Standardized amorphous Goodfellow PET film (GfPET, ES30-FM-000145, Goodfellow Cambridge Ltd., UK) was cut into strips (∼2.5 × 0.6 cm) weighing 45 mg each and washed with ethanol and reverse osmosis (RO) water, dried, and transferred to 2 mL reaction tubes. To each tube, potassium phosphate buffer (pH 8, either 0.1 or 1 M) and purified enzyme stock were added in a final volume of 1.8 mL. The assays were incubated at either 65 or 70 °C and 700 rpm in a thermal shaker (VWR thermal shaker lite) for 16 h. Subsequently, the films were removed and washed in RO water, 0.5% SDS, RO water, and 70% ethanol before drying at 37 °C and weighing. The start and end weights of the PET films were used to calculate the percentage of PET degraded in 16 h.

### Postconsumer Thermoform PET Degradation Assays

Enzymatic PET degradation in a 0.5 L scale was performed using postconsumer thermoform packaging (R-PET clamshells), which was cut into flakes of ∼1.5 × 1.5 cm. The experiments were conducted in a double-walled (jacketed) flat-bottom cylindrical reaction vessel (max. working volume of 0.7 L) to maintain a constant temperature of 62.5 °C, controlled by a thermostat. The reactor was filled with 0.5 L of 0.1 M potassium phosphate buffer and 50 g postconsumer thermoform PET flakes (100 g L^–1^). To start the reaction, 40 mg of enzyme was added. The mixture was constantly stirred at 200 rpm. The pH was maintained at pH 8.0 by dosing 5 M NaOH with a pH probe and control software. The NaOH reservoir was placed on a balance, allowing real-time gravimetric measurement of NaOH consumption. As PET is hydrolyzed, terephthalic acid (TPA) is released, which lowers the pH; dosing of NaOH neutralizes the acid and thus maintains constant pH. The amount of NaOH added can be stoichiometrically correlated to the amount of PET degraded, as each mole of TPA released consumes 2 mol of NaOH. The mass of PET degraded (in g/L^−1^) can be calculated using the following formula:

$$PET\;degraded\;\left[g/L^{-1}\right]=\frac{m_{NaOH\;Sol.}\;\left[g\right]\times c_{NaOH\;Sol.}\;\left[mol/L\right]\times M_{PET}\;\left[g/mol\right]}{2\times \rho_{NaOH}\;g/ml\times 1000\times V\left[L\right]}$$

where *c*_NaOH_ = 5 M, ρ_NaOH_ = 1.185 g/mL^−1^, and *M*_PET_ = 192.2 g/mol.

## Results

### Enzyme Sequences of Polyester Hydrolysis Variants

In this study, the PHL7 wild-type enzyme and the improved PHL7 triple mutant PHL7mut3 were expressed in *E. coli* and *P. pastoris*, and their PET-degrading activity under various conditions as well as their thermal stability was assessed. Both the PHL7 and the PHL7mut3 sequences contain three putative N-glycosylation sites (N143, N144, and N161). To assess whether the enzymatic properties of the *E. coli*-produced enzymes can be reproduced when *P. pastoris* is used as a host, non-N-glycosylated enzyme variants were designed by substituting the asparagine (N) residues at the N-glycosylation sites with glutamine (Q) residues (Supporting Information, S1). Additionally, O-glycosylations may also occur in *P. pastoris*. However, it is notoriously difficult to predict O-glycosylation due to the lack of a strict consensus sequence and the variability introduced by different production conditions.^33^ In total, six enzymes, two sets of three variants each, were produced and purified (Table 1). The amino acid sequences of E_PHL7 and P_PHL7 are identical with the exception of a short “LE” linker, which is present in E_PHL7 before the His-tag.

**Table 1:** Polyester Hydrolases and Production Hosts Used in This Study

enzyme	production host	N-glycosylation	abbreviation
PHL7	*E. coli*	no	E_PHL7
PHL7	*P. pastoris*	yes	P_PHL7
PHL7	*P. pastoris*	no (N143Q/N144Q/N161Q)	P_PHL7_ng
PHL7mut3	*E. coli*	no	E_PHL7mut3
PHL7mut3	*P. pastoris*	yes	P_PHL7mut3
PHL7mut3	*P. pastoris*	no (N143Q/N144Q/N161Q)	P_PHL7mut3_ng

### Expression and Purification of PHL7 and PHL7mut3 Variants

All enzymes were expressed with a C-terminal His-tag and purified by using Immobilized Metal Affinity Chromatography. No host cell proteins were detected in the purified enzyme samples (Figure 1A). P_PHL7 and P_PHL7mut3 (lane 6 and lane 9, respectively) showed more than one band and a smear at higher molecular weights. This is typically observed for glycosylated proteins. It is noticeable that the bands of P_PHL7_ng and P_PHL7mut3_ng appeared at slightly higher apparent molecular masses than their *E. coli* produced counterparts E_PHL7 and E_PHL7mut3.

**Figure 1:**
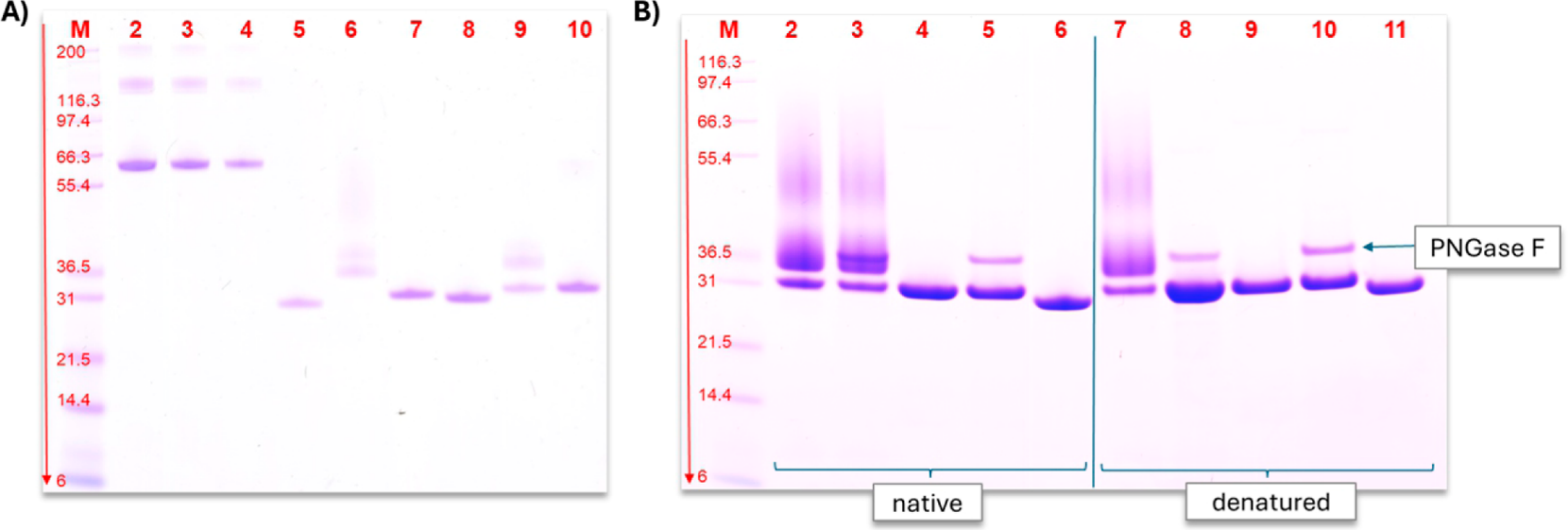
SDS-PAGE analysis of the purified and PNGase F-treated enzymes. A: purified enzymes, lane M: Thermo Scientific Invitrogen Mark 12 unstained standard, 2.5–200 kDa, lanes 2–4: bovine serum albumin standards with 75, 50, and 25 μg mL^–1^, lane 5: E_PHL7 (*E. coli* produced), lane 6: P_PHL7, lane 7: P_PHL7_ng, lane 8: E_PHL7mut3, lane 9: P_PHL7mut3, and lane 10: P_PHL7mut3_ng. B: PHL7 variants treated with PNGase F for deglycosylation, lane M: Thermo Scientific Invitrogen Mark 12 unstained standard, 2.5–200 kDa, lanes 2 + 7: P_PHL7, lanes 3 + 8: P_PHL7 + PNGase F, lanes 4 + 9: P_PHL7_ng, lanes 5 + 10: P_PHL7_ng + PNGase F, lanes 6 + 11: E_PHL7, lanes 2–6: enzymes treated with PNGase F while still in native form, and lanes 7–11: enzymes treated with PNGase F after denaturation.

SDS-PAGE analysis (Figure 1B) confirmed the successful production of PHL7 variants without N-glycosylation (P_PHL7_ng) in *P. pastoris*. In the native samples (lanes 2–6), N-glycosylation remained unaffected by PNGase F treatment (lane 3), as the carbohydrate structures were likely inaccessible in the folded protein. After denaturation (lanes 7–11), PNGase F treatment (lane 8) completely removed N-glycosylations, while P_PHL7_ng (lane 10) showed no change in band position, confirming the absence of N-glycosylations.

### Temperature Stability of PHL7 and PHL7mut3 Variants

The onset of denaturation and the melting point of polyester hydrolases are critical parameters for their application, as PET hydrolysis is typically performed at around 60–70 °C in the glass transition temperature range of PET. If the denaturation onset is lower than the assay temperature, a rapid loss of enzyme activity over time is expected. For PHL7, only the N-glycosylated variant (P_PHL7) showed an onset of denaturation above 70 °C (Table 2). In contrast, the non-glycosylated variant (P_PHL7_ng) had a lower onset temperature of 65 °C, which is slightly below that of E_PHL7 (67 °C). For PHL7mut3, the variants followed the same trend, with P_PHL7mut3 having the highest onset of denaturation at 75 °C, followed by E_PHL7mut3 at 73.6 °C and P_PHL7mut3_ng at 68.5 °C. Overall, the PHL7mut3 variants showed higher melting points and onsets of denaturation than their PHL7 counterparts. Interestingly, the difference in the onset of denaturation was much more pronounced between E_PHL7 and P_PHL7 at 5.5 °C than between E_PHL7mut3 and P_PHL7_mut3 at only 1.4 °C. The difference between the onset of denaturation and the inflection point consistently ranged from 8 to 9 °C for all enzyme variants, except for PHL7 produced in *E. coli* at 12 °C.

**Table 2:** Onset of Denaturation and Inflection Point of PHL7 and PHL7mut3 Produced in *E. coli* (E) and *P. pastoris* (P) Measured with NanoDSF^a^ ^,^ ^b^

	onset of denaturation (*T*_on_)		inflection point (*T*_m_)	
enzyme	temp. [°C]	SD [°C]	temp. [°C]	SD [°C]
E_PHL7	67.0	0.7	79.3	0.0
P_PHL7	72.5	0.1	81.2	0.1
P_PHL7_ng	65.2	0.6	74.4	0.0
E_PHL7mut3	73.6	0.1	82.7	0.0
P_PHL7mut3	75.0	0.2	83.2	0.0
P_PHL7mut3_ng	68.5	0.3	77.6	0.2

a Melting temperatures of all six enzymes in 1 M potassium phosphate buffer are provided in Supporting Information, S2.

b Mean values for *n* = 3 experiments ±SD are shown.

### Investigation of Glycosylation and Enzyme Structure

The influence of N-glycosylation on the enzyme activity is difficult to predict because many different conformations for the glycan structures are possible. All modeled conformations for the N-glycans attached at N143 and N161 were visualized (Figure 2), assuming Man9GlcNAc2 glycans (see Supporting Information, S3). As the Nx­(ST) sequons for N143 and N144 are overlapping and literature suggests glycosylation in NNTS motifs almost exclusively occurs at the first Asn residue,^34^ N144 was not included as a glycosylation site in the model. None of the predicted conformations directly obstructed the active site of the enzyme. While glycosylation may still impact enzyme activity through factors such as steric hindrance, a complete loss of activity is unlikely.

**Figure 2:**
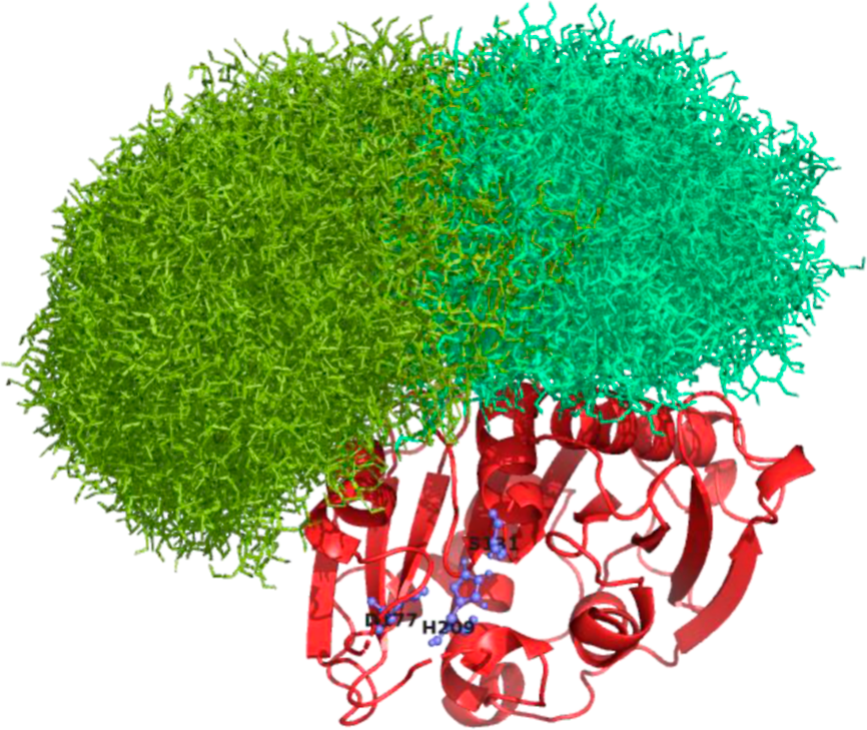
AlphaFold 3 structure predictions (visualized in PyMOL) of PHL7 enzyme (red structure), green structures show all possible conformations of Man_9_GlcNAc_2_ N-glycosylations at positions N143 and N161 modeled according to the method developed by Turupcu et al.^26^ The amino acids highlighted in blue (S131, D177, and H209) are the catalytic triad of PHL7.

To assess the impact of mutations and glycosylation on structural folding, we used X-ray crystallography to determine the structures of PHL7mut3, expressed in both *E. coli* (E_PHL7mut3) and *P. pastoris* (P_PHL7mut3), as well as a non-glycosylated variant with three additional mutations (P_PHL7mut3_ng), also expressed in *P. pastoris*.

The three proteins crystallized in different crystal forms. The E_PHL7mut3 crystal belonged to the *C*2 space group, while the P_PHL7mut3 and P_PHL7mut3_ng crystals belonged to the *P*2_1_2_1_2_1_ space group. This complicated the comparison of the crystal structures, as differences in crystal packing have to be considered in addition to the differences in glycosylation or other modifications.

The crystal structures were resolved at atomic resolution with the highest resolution of 0.89 Å achieved for E_PHL7mut3. Data for P_PHL7mut3_ng and P_PHL7mut3 were collected to resolutions of 1.02 and 1.37 Å, respectively. Structure determination revealed that P_PHL7mut3 and P_PHL7mut3_ng crystallized with one molecule per asymmetric unit, whereas E_PHL7mut3 contained two molecules in the asymmetric unit. The high-resolution structures demonstrated an almost identical overall fold to that of the PHL7 wild type (PDB ID: 7NEI), characterized by an α/β hydrolase fold with a catalytic triad comprising Ser-Asp-His.^8^ Secondary structural elements, including α-helices and β-sheets, align precisely with the wild-type backbone, with minor deviations observed in flexible loop regions (Figure 3A).

**Figure 3:**
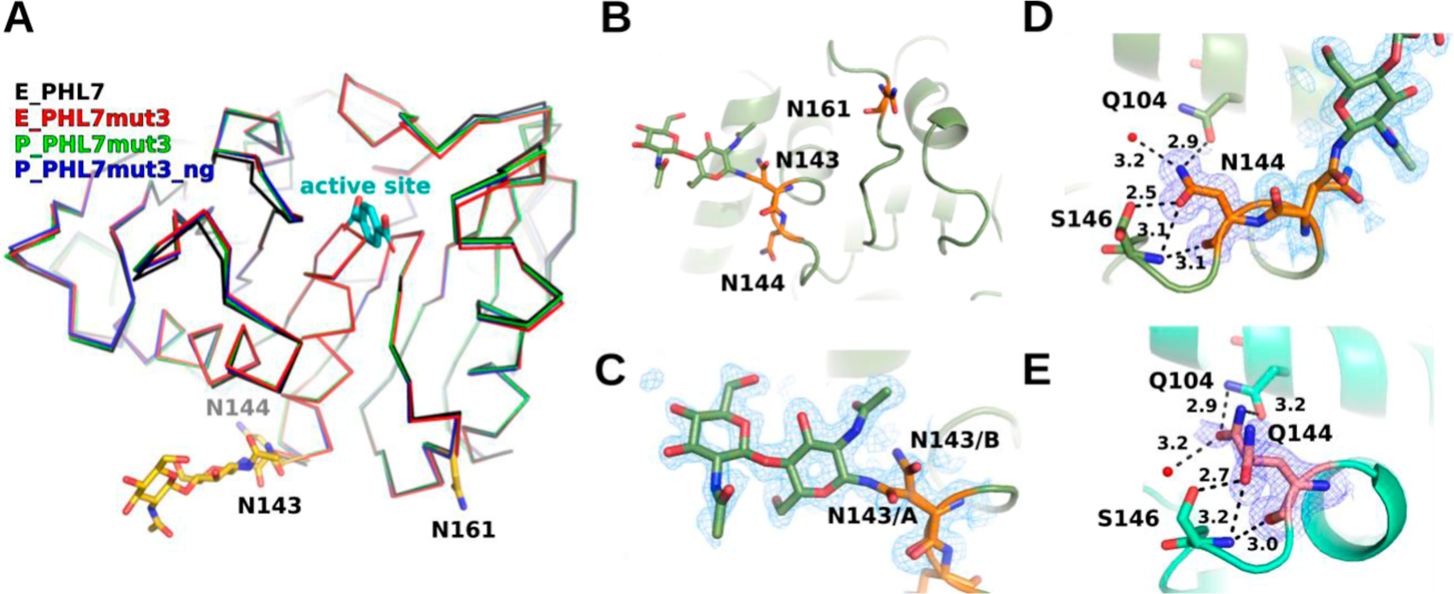
(A) Superposition of E_PHL7mut3 (red), P_PHL7mut3 (dark green), P_PHL7mut3_ng (blue), and wildtype E_PHL7 (gray, pdb id 7NEI). The TPA product (TPA, cyan) has been superimposed from the PHL7 × TPA cocrystal structure (pdb id 8bra) to mark the active site. (B) Two N-acetylglucosamine residues were modeled at N143 in P_PHL7mut3. (C) Electron density map (2Fo-Fc map contoured at 0.7 σ_rmsd_) of the N143 glycosylation in P_PHL7. (D) Hydrogen bonding interaction of N144 (2Fo-Fc map at 1.0 σ_rmsd_) in P_PHL7. (E) Two alternative conformations of Q144 in P_PHL7mut3_ng and 2Fo-Fc-type map contoured at 0.7 σ_rmsd_.

For P_PHL7mut3, the glycosylation of N143 was observed (Figure 3B). Two N-acetyl-glucosamine residues could be modeled, but not the additional residues of the glycan chain, likely due to disorder (Figure 3C). The electron density for residues N161 and N144 showed no evidence of glycosylation. N143 adopted two distinct conformations, with glycan attachment observed only in conformation A. The occupancy was refined to 0.72 for the first N-acetyl glucosamine unit and 0.56 for the second. The B-factors for the glycan were comparable to those of the surrounding residues, with a slightly elevated B-factor for the second mannose unit, indicating increased flexibility. The electron density for residues N161 and N144 showed no evidence of glycosylation. The electron density map does not indicate O-glycosylation at any of the Ser or Thr residues.

PHL7mut3 has been designed to improve the activity and thermostability of PHL7 by the Q175E, L210T, and D233 K mutations. Structural comparison of the mutation sites Q175E and L210T revealed no differences in backbone or side-chain conformation among the three PHL7mut3 structures (Figure 4A,B,D,E). However, the K233 residue exhibited distinct conformational variability (Figure 4C). In the P_PHL7mut3 structure, electron density supported a single conformation for K233 (Figure 4F), whereas two conformations were modeled for the same residue in E_PHL7mut3 and P_PHL7mut3_ng. Very weak density indicates multiple conformations of K233 beyond the C_γ_-atom in these two structures (Figure 4C). The single conformation of K233 observed in the P_PHL7mut3 structure is supported by a well-defined water network at a crystal contact (Figures 4F and S5B). We therefore consider the observed flexibility of K233 in the other two structures as characteristic for the enzyme in solution.

**Figure 4:**
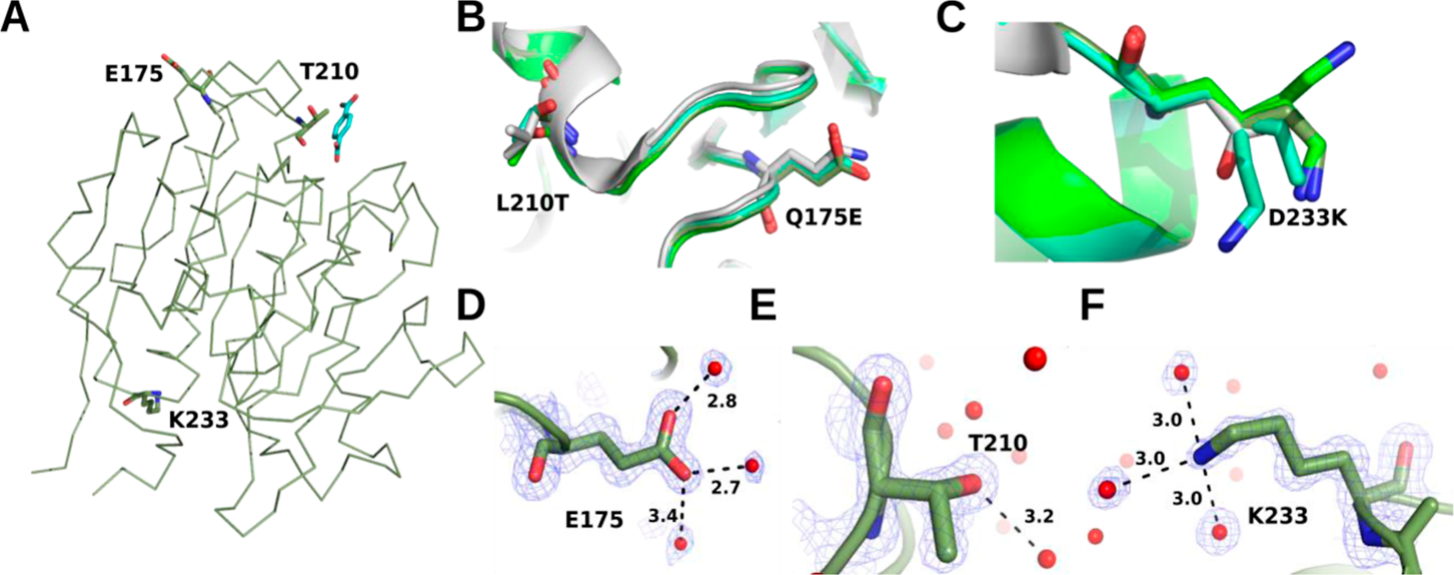
Analysis of the PHL7mut3 mutations Q175E, L210T, and D233 K, which increase the thermostability of PHL7. (A) Overall structural fold of E_PHL7mut3 with the three mutations displayed in sticks, and the active site represented by terephthalic acid (cyan), which has been superimposed from the PHL7 × TPA cocrystal structure (pdb id 8bra) to mark the active site. (B,C) Superposition of the mutated residues in E_PHL7mut3 (green), P_PHL7mut3_ng (cyan), P_PHL7mut3 (dark green), and the wildtype PHL7 (gray). (D–F) Interactions of E175 (D), T210 (E), and K233 (F) of P_PHL7mut3. Water molecules are represented as red spheres and distances to water molecules are given in Angstrom (D–F). The 2Fo-Fc-type electron density maps are contoured at 1.0 σ_rmsd_.

### Amorphous PET Film Degradation in 1 M Potassium Phosphate Buffer

GfPET films were hydrolyzed at 65 and 70 °C with different concentrations of PHL7 produced in *E. coli* and in *P. pastoris*, with and without N-glycosylations, in 1 M potassium phosphate buffer (Figure 5). This experiment aimed to compare the activity of enzyme variants produced in *E. coli* and *P. pastoris* and to evaluate how PHL7 and PHL7mut3 expressed in *P. pastoris* without N-glycosylation perform relative to E_PHL7 and E_PHL7mut3.

**Figure 5:**
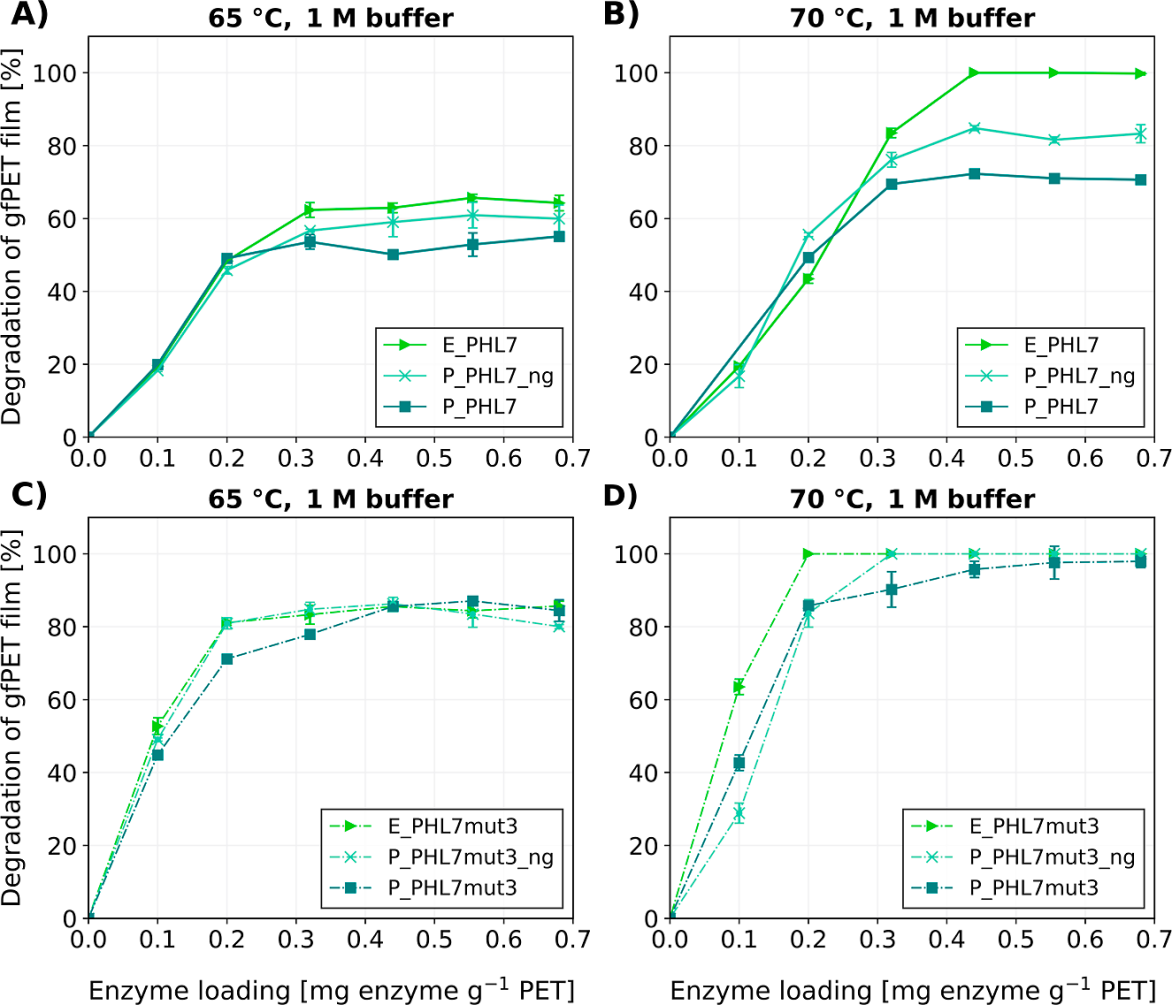
Comparison of amorphous PET film degradation at different enzyme loadings (0.1, 0.2, 0.32, 0.44, 0.56, and 0.68 mg enzyme g^–1^ PET) within 16 h in 1 M potassium phosphate (KPO) buffer with PHL7 (A + B) or PHLmut3 (C + D) produced in *E. coli*, P. pastoris, and P. pastoris without N-glycosylation; (A + C) at 65 °C incubation temperature and (B + D) at 70 °C incubation temperature. Mean values for *n* = 3 experiments ±SD are shown.

At 65 °C, the maximum degradation of the PET films for the PHL7 variants (Figure 5A) after 16 h of reaction was 52%, 60%, and 65% for P_PHL7, P_PHL7_ng, and E_PHL7, respectively. At 70 °C, all PHL7 enzymes showed significantly higher activity (Figure 5B). E_PHL7 completely degraded the films after 16 h. P_PHL7 and P_PHL7_ng achieved 70% and 82% degradation, respectively. While there only is a slight difference in activity between the PHL7 enzyme variants at 65 °C, the effect is more pronounced at 70 °C. For the PHL7mut3 enzyme variants, the difference in PET degradation performance was less pronounced compared to the PHL7 enzymes. At 65 °C, E_PHL7mut3, P_PHL7mut3, and P_PHL7mut3_ng performed nearly identical at all enzyme loadings (Figure 5C) with a maximum of 85% of the PET film hydrolyzed in 16 h. At 70 °C (Figure 5D), E_PHL7mut3 and P_PHL7mut3_ng reached complete degradation in 16 h at 0.2 and 0.32 mg of enzyme g^–1^ PET, respectively. P_PHL7mut3 approached full degradation at high enzyme loadings but never reached 100%. At enzyme loadings below 0.2 mg enzyme g^–1^ PET, P_PHL7mut3 and P_PHL7mut3_ng both similarly showed slightly lower activities than E_PHL7mut3. Higher enzyme concentrations resulted in a linear increase in activity, until a plateau of enzyme saturation was reached.

### Amorphous PET Film Degradation in 0.1 M Potassium Phosphate Buffer

Initial tests showed the highest enzyme stability in a 1 M potassium phosphate buffer. While such high salt concentrations are acceptable for small-scale assays, they may not be economically feasible for industrial use. To explore lower buffer concentrations, PET degradation was tested in 0.1 M buffer (Figure 6) with two enzyme loadings: 0.1 mg enzyme g^–1^ PET (below saturation for all variants) and 0.44 mg enzyme g^–1^ PET (saturation of the PET film surface).

**Figure 6:**
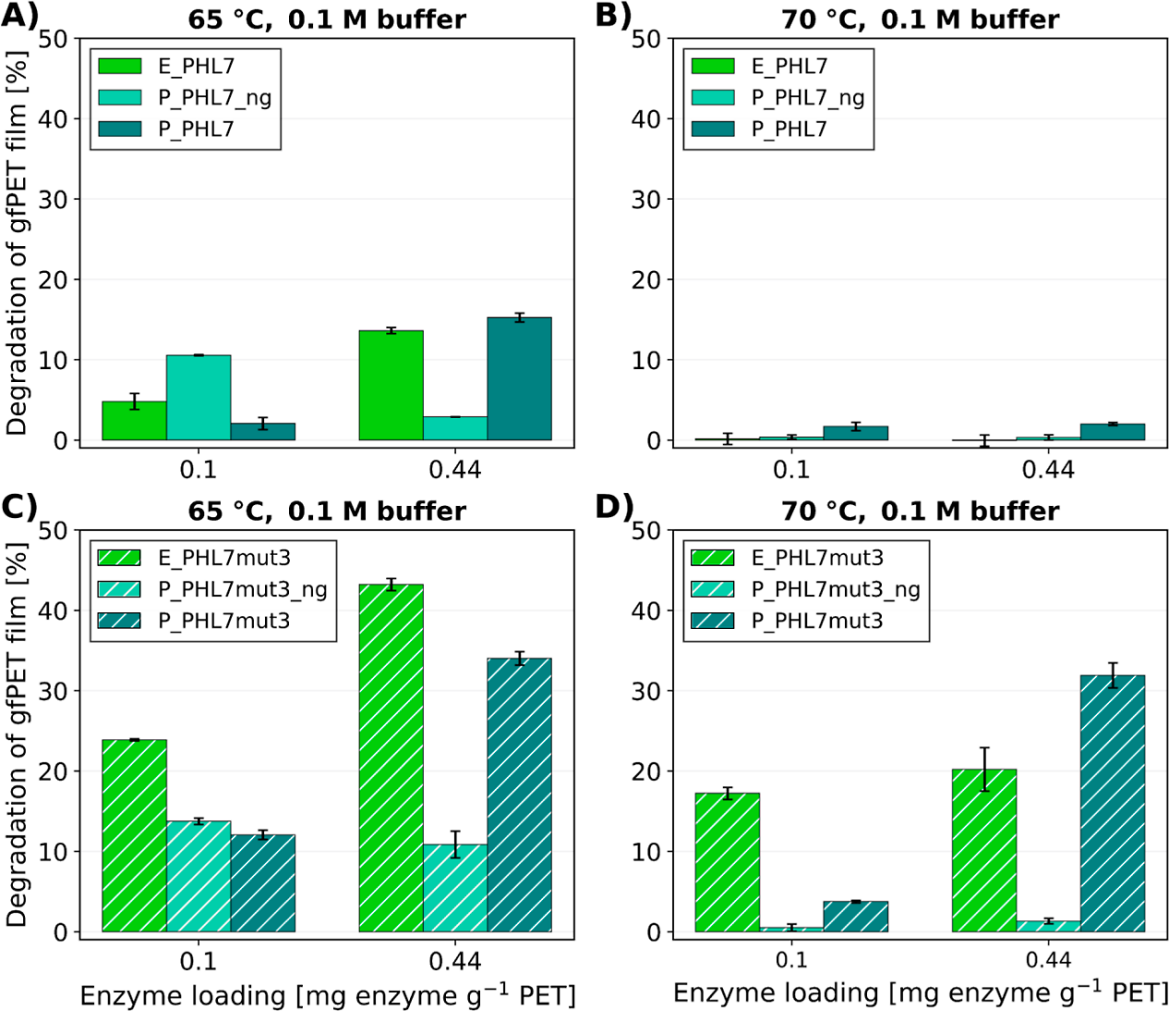
Comparison of amorphous PET film degradation at enzyme loadings of 0.1 and 0.44 mg enzyme g^–1^ PET in 16 h in 0.1 M potassium phosphate buffer with PHL7 (A + B) or PHL7mut3 (C + D) produced in *E. coli*, *P. pastoris*, and *P. pastoris* without N-glycosylation; (A + C) at 65 °C incubation temperature and (B + D) at 70 °C incubation temperature. Mean values for *n* = 3 experiments ±SD are shown.

For all enzymes, a lower PET degradation was observed in 0.1 M buffer compared with 1 M buffer. In contrast to the experiments carried out in 1 M buffer, the degradation of PET films was higher at 65 °C than at 70 °C in 0.1 M buffer. For the PHL7 variants, the highest PET degradation was observed at 65 °C with 14 and 15% degradation within 16 h for E_PHL7 and P_PHL7, respectively. At 70 °C, E_PHL7 and P_PHL7_ng showed no PET degradation, and P_PHL7 achieved 2% weight loss. The PHL7mut3 variants showed a higher PET-degrading performance than the PHL7 enzymes at both temperatures. At 70 °C, the highest PET degradation was achieved by P_PHL7_mut3 with 34%. At 65 °C, E_PHL7mut3 reached 43% in 16 h.

### Degradation of Postconsumer Thermoform PET by PHL7mut3 Variants

Subsequently, we compared the performance of the PHL7mut3 enzymes in the degradation of postconsumer PET material. The hydrolysis of thermoform PET flakes was performed in a 0.5 L reactor using 0.1 M potassium phosphate buffer. The reactor was loaded with 100 g L^–1^ PET, and the degradation at 62.5 °C was measured over the course of 48 h (Figure 7). The initial slope of E_PHL7mut3 and P_PHL7mut3, and therefore their PET degradation rate, is identical, but starts to diverge after 5 h. In contrast, P_PHL7mut3_ng demonstrates a significantly lower initial slope, indicating a slower rate of PET degradation. While the degradation curves of P_PHL7mut3 and P_PHL7mut3_ng start to run parallel after about 10 h, the curve of E_PHL7mut3 runs more steeply and consequently diverges from the other two curves to a greater extent over time. Within 48 h, the total amounts of PET degraded by E_PHL7mut3, P_PHL7mut3, and P_PHL7mut3_ng were 77.3 g L^–1^, 63.3 g L^–1^, and 53.9 g L^–1^, respectively.

**Figure 7:**
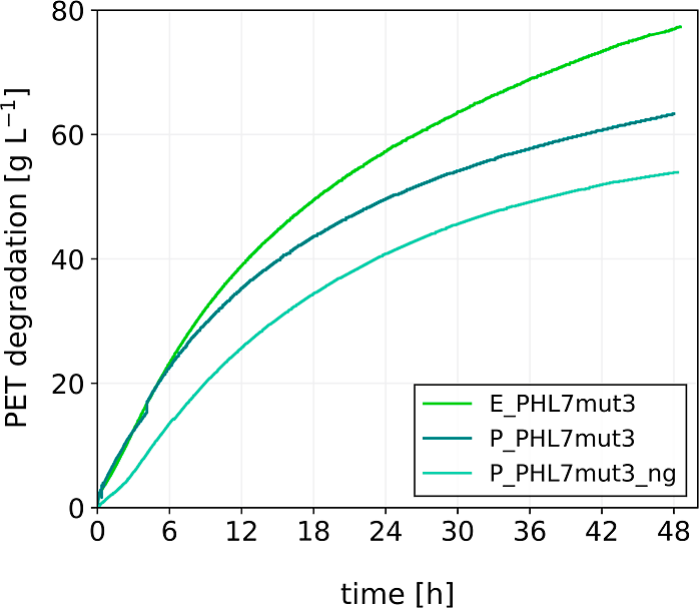
Degradation of postconsumer thermoform PET by 0.8 mg PHL7mut3 per gram PET using enzyme produced in *E. coli* and *P. pastoris* (glycosylated and non-glycosylated) at 62.5 °C and pH 8 in 0.1 M potassium phosphate buffer; single runs measured in real-time through NaOH consumption.

## Discussion

### Denaturation Temperatures of PHL7 and PHL7mut3 Variants

The enzymatic degradation of PET was so far found to be most effective at temperatures in the range of 60–70 °C, which coincides with the onset of its glass transition.^35–37^ In this temperature range, the PET polymer chains become more flexible, rendering the ester bonds more accessible to enzymatic cleavage. Therefore, an onset of enzyme denaturation above 70 °C ensures enzyme stability throughout the reaction time, while promoting efficient PET hydrolysis.^38,39^ N-glycosylation is known to enhance thermal stability of proteins,^40^ which was also observed in the current study for both PHL7 and PHL7mut3. The mutations introduced to obtain non-glycosylated enzymes in *P. pastoris* resulted in a decrease in melting temperature compared to the *E. coli-*produced enzymes. The consistent difference of 8–9 °C between the onset of denaturation and the inflection point (*T*_m_) observed for most variants reflects the cooperative nature of the unfolding transition, with the broader transition in *E. coli*-produced PHL7 (12 °C difference), suggesting a less uniform or less stable unfolding behavior compared to the other enzyme preparations.

### Glycosylation of PHL7 and PHL7mut3

To accurately compare the enzymatic activities, purified enzyme preparations were used to ensure that observed differences were due to intrinsic properties without influence from other compounds in the fermentation medium. As shown in Figure 1, the enzyme purification process yielded high-purity enzymes, as evidenced by a well-defined single band on SDS-PAGE for the non-glycosylated variants. Glycosylated variants exhibited a characteristic smear, indicating heterogeneity in glycosylation, consistent with the presence of various glyco-forms.^41,42^ The absence of additional well-defined bands indicated pure glycosylated P_PHL7 and P_PHL7mut3. To further confirm the absence of N-glycosylation in the N143Q, N144Q, and N161Q enzyme variants, PNGase F treatment was employed (Figure 1B). Only after denaturation, this treatment effectively removed N-glycans from the enzyme, suggesting that the bond between the asparagine and the first N-acetyl glucosamine in the natively folded protein is not easily accessible to PNGase F. PNGase F-treated P_PHL7 migrated as a single discrete band, contrasting untreated P_PHL7, whereas both PNGase F-treated and untreated P_PHL7_ng looked the same. This indicates that the glycans in P_PHL7 were successfully removed by the PNGase F treatment, while P_PHL7_ng does not contain N-glycosylations that could have been cleaved by PNGase F. Slightly higher apparent molecular weights of *P. pastoris*-produced enzymes (both glycosylated and non-glycosylated) on SDS-PAGE gels in comparison to the *E. coli-*produced variants, even after PNGase F treatment, may result from subtle PTMs, such as phosphorylation, acetylation, or methylation, or from interactions with host-derived components.

### Investigation of Glycosylation and Enzyme Structure

Figure 2 shows computational modeling of all possible confirmations of the modeled Man_9_GlcNAc_2_ structures at the N143 and N161 sites based on free-energy landscapes.^26^ These models indicate that the N-glycans do not block the active site directly, implying that the enzyme activity is not likely to be impaired by steric hindrance at the catalytic site. However, the bulky and polar nature of the glycans might still impede substrate binding or cause changes in enzyme polarity, affecting interactions with the apolar PET surface.^43^ Additionally, glycosylation could increase the rigidity of the enzymes, potentially reducing their conformational flexibility and slowing catalytic conversion. We hypothesized that eliminating glycosylation could be beneficial for enzyme activity. To test this, we engineered enzyme variants without N-glycosylation sites by substituting the corresponding asparagine residues with glutamine, resulting in three equilibrated amino acids.

We determined crystal structures of E_PHL7mut3, P_PHL7mut3, and P_PHL7mut3_ng to characterize possible structural differences resulting from the glycosylations. Of the three putative N-glycosylation sites N143, N144, and N161, only N143 was found to be glycosylated in the crystal structure of P_PHL7mut3 with an occupancy of 0.72. Due to the proximity of N143 and N144, steric hindrance likely prevented glycosylation at both sites, suggesting each enzyme was glycosylated at no more than two sites, which was also described by Reddy et al.^34^ The well-defined electron densities of N144 and N161 suggest that these residues are not attached to glycan chains in the crystal. N161 is involved in crystal contact with residue H264, the penultimate histidine residue of the His-tag of this construct. It is thus conceivable that a fraction of the protein in the crystallization mixture was glycosylated at N161, but only the N161-unglycosylated fraction was enriched in the crystal because glycosylated N161 is not compatible with this crystal form. N144 is more buried than N143 and it forms hydrogen bridges to Q104 and S146 with its N_δ2_ and O_δ1_ atoms, respectively (Figure 3D). The buried position of N144 might prevent its glycosylation. O-glycosylation was not found at any of the Ser or Thr residues according to the electron density map. However, it is important to consider that the crystallization process can preferentially select non-glycosylated protein from a heterogeneously glycosylated sample, particularly if the glycosylation site is located near a crystal contact.

Superposition of the three crystallized variants revealed no significant differences that could not be attributed to crystal contacts. The N143 glycosylation site is approximately 17 Å away from the substrate binding site, but it is part of the loop that links an α-helix and a β-strand forming part of the active site structure (Figure S5A). Thus, glycosylation at N143 might influence the dynamics of the active site. The well-defined 1.37 Å electron density map of P_PHL7mut3 revealed no covalent modifications that might explain the observed differences in catalytic properties in addition to the glycosylation. The difference in the catalytic properties of E_PHL7mut3 and P_PHL7mut3_ng might be caused by the N143Q, N144Q, and N161Q mutations. Whereas N143 and N161 are highly solvent exposed, and mutation to a glutamine likely has little influence on PHL7 structure and dynamics; the N144Q mutation destroys the hydrogen-bonding interactions of the N144 side chain carboxamide group with Q104 and S146 (Figure 3E) because of steric hindrances. This difference might be primarily responsible for the observed catalytic differences between E_PHL7mut3 and P_PHL7mut3_ng. Assuming there are no differences in PTM between these two variants, they differ only in the three asparagine to glutamine mutations and in the presence of an additional leucine-glutamate linker N-terminal to the His-tag in the *E. coli*-produced enzymes. The C-terminus is quite solvent-exposed, located at a distance of >35 Å to the active site at the opposite side of the protein. Most likely, the additional LE-linker has little influence on the catalytic activity of PHL7.

### PET Degradation Performance of PHL7 and PHL7mut3 Variants

In 1 M potassium phosphate buffer, the *E. coli*-expressed enzymes E_PHL7 and E_PHL7mut3 exhibited the highest PET degradation activity, followed by the non-glycosylated *P. pastoris*-produced variants, with the glycosylated enzymes showing the lowest degradation. This trend was most pronounced at 70 °C with wild-type PHL7. The least difference between the variants was found at 65 °C with the PHL7mut3 enzymes. The reduced activity of the *P. pastoris*-expressed variants might be attributed to an increased rigidity and steric hindrance due to the glycosylation.^17–19,44^ The non-glycosylated *P. pastoris* variants, while more active than their glycosylated counterparts, did not match the activity of the *E. coli*-expressed enzymes. The altered enzyme characteristics may be caused by the N-to-Q mutations or differences in protein folding and processing in *P. pastoris*.^45,46^ The lower melting point of the non-glycosylated *P. pastoris*-produced variants likely plays a role in their reduced PET degradation performance. The reasons for the less pronounced difference in activity between the variants at 65 °C compared to 70 °C, particularly with PHL7mut3, remain unclear and warrant further investigation.

In 0.1 M potassium phosphate buffer, the performance of all enzyme variants decreased compared to that in 1 M buffer. The lowest activity was observed with PHL7 variants at 70 °C, while the combination of PHL7mut3 variants and 65 °C showed the best performance in low buffer concentrations. This suggests a temperature stability issue in lower buffer concentrations, as supported by the work of Pfaff et al. on PES-H1 (syn. PHL7).^47^ Among all variants tested, at 65 °C, E_PHL7mut3 exhibited the best performance in PET hydrolysis. At lower enzyme loadings, enzymes produced in *P. pastoris* consistently showed a lower degradation performance compared to their *E. coli*-produced counterparts. One possible explanation is that glycan structures introduced by glycosylation may partially hinder substrate binding or reduce surface adsorption efficiency, especially on a solid polymer surface like PET. This steric effect could require higher enzyme concentrations to achieve sufficient surface coverage or effective catalytic engagement. Once surface saturation is reached, the negative effect of glycosylation appears less pronounced, possibly because excess enzyme compensates for a subset of molecules that are sterically hindered by their specific glycan structures. This highlights that, for the P_PHL7mut3 enzyme, the increased thermostability conferred by N-glycosylation does not compensate for the negative impact on its activity, especially at low enzyme loadings.

In the pH-controlled 0.5 L degradation experiments using 0.1 M buffer and postconsumer PET at 62.5 °C, the PHL7mut3 variants followed the same trend observed in the small-scale assays. Maintaining pH control during the reaction mitigated the negative effects of acidification due to TPA release, allowing for a more accurate assessment of enzyme performance. The highest amount of hydrolyzed PET was achieved with E_PHL7mut3, followed by P_PHL7mut3, and last P_PHL7mut3_ng.

## Conclusion

The PHL7 enzyme attracted attention for its rapid PET degradation performance. PHL7 exhibits its highest activity at 70 °C but requires high salt concentrations for stability at this temperature. Achieving temperature stability at low salt concentrations is desirable for practical applications. Previous studies on PET-hydrolyzing enzymes have indicated that glycosylation can enhance the temperature stability and potentially improve activity. To obtain glycosylated enzymes, we expressed PHL7 in *P. pastoris*, and crystallography revealed certain N-glycosylation at one of the three putative N-glycosylation sites. This glycosylation improved the temperature stability but resulted in decreased enzyme activity.

Based on the outcome of this study, engineering the enzyme for improved temperature stability, as demonstrated with PHL7mut3, generally yielded a more favorable PET degradation performance than producing glycosylated variants. Additionally, slightly lowering the process temperature could balance the trade-off between stability and activity. The *P. pastoris*-produced variants without N-glycosylation sites exhibited reduced temperature stability and activity compared to the *E. coli*-produced enzymes. Therefore, expressing PHL7mut3 in host organisms that do not add glycosylation is advisable, provided that the same product titer can be achieved.

## Supplementary Material



## Notes

Amino acid sequences, melting temperatures of enzyme variants in 1 M potassium phosphate buffer, structure of mannose-9 N-linked oligosaccharide used for modeling, diffraction data, and crystal structure of P_PHL7mut3 (PDF)

## References

[1] Geyer R., Jambeck J. R., Law K. L. (2017). Production, use, and fate of all
plastics ever made. Sci. Adv..

[2] Lebreton L. C. M., Van Der Zwet J., Damsteeg J. W., Slat B., Andrady A., Reisser J. (2017). River plastic
emissions to the world’s oceans. Nat.
Commun..

[3] Jambeck J. R. (2015). Plastic waste inputs
from land into the ocean. Science.

[4] Sui B., Wang T., Fang J., Hou Z., Shu T., Lu Z., Liu F., Zhu Y. (2023). Recent advances
in the biodegradation
of polyethylene terephthalate with cutinase-like enzymes. Front. Microbiol..

[5] Müller R., Schrader H., Profe J., Dresler K., Deckwer W. D. (2005). Enzymatic
degradation of poly­(ethylene terephthalate): Rapid hydrolyse using
a hydrolase from T. fusca. Macromol. Rapid Commun..

[6] Tournier V., Duquesne S., Guillamot F., Cramail H., Taton D., Marty A., André I. (2023). Enzymes’
Power for Plastics
Degradation. Chem. Rev..

[7] Sulaiman S. (2012). Isolation of a novel cutinase homolog with
polyethylene terephthalate-degrading
activity from leaf-branch compost by using a metagenomic approach. Appl. Environ. Microbiol..

[8] Sonnendecker C., Oeser J., Richter P. K., Hille P., Zhao Z., Fischer C., Lippold H., Blázquez-Sánchez P., Engelberger F., Ramírez-Sarmiento C. A. (2022). Low
Carbon Footprint Recycling of Post-Consumer PET Plastic with a Metagenomic
Polyester Hydrolase. ChemSusChem.

[9] Tournier V. (2020). An engineered PET depolymerase to break down
and recycle plastic
bottles. Nature.

[10] Zheng Y., Li Q., Liu P., Yuan Y., Dian L., Wang Q., Liang Q., Su T., Qi Q. (2024). Dynamic Docking-Assisted
Engineering of Hydrolases for Efficient PET Depolymerization. ACS Catal..

[11] Cui Y., Chen Y., Sun J., Zhu T., Pang H., Li C., Geng W. C., Wu B. (2024). Computational redesign of a hydrolase
for nearly complete PET depolymerization at industrially relevant
high-solids loading. Nat. Commun..

[12] Richter P. K., Blázquez-Sánchez P., Zhao Z., Engelberger F., Wiebeler C., Künze G., Frank R., Krinke D., Frezzotti E., Lihanova Y. (2023). Structure and function
of the metagenomic plastic-degrading polyester hydrolase PHL7 bound
to its product. Nat. Commun..

[13] Fohler L., Leibetseder L., Cserjan-Puschmann M., Striedner G. (2024). Manufacturing
of the highly active thermophile PETases PHL7 and PHL7mut3 using Escherichia
coli. Microb. Cell Fact..

[14] Shirke A. N., White C., Englaender J. A., Zwarycz A., Butterfoss G. L., Linhardt R. J., Gross R. A. (2018). Stabilizing
Leaf and Branch Compost
Cutinase (LCC) with Glycosylation: Mechanism and Effect on PET Hydrolysis. Biochemistry.

[15] Rosano G. L., Ceccarelli E. A. (2014). Recombinant protein expression in
Escherichia coli:
Advances and challenges. Front. Microbiol..

[16] Su L., Xu C., Woodard R. W., Chen J., Wu J. (2013). A novel strategy
for
enhancing extracellular secretion of recombinant proteins in Escherichia
coli. Appl. Microbiol. Biotechnol..

[17] Wang Z., Guo C., Liu L., Huang H. (2018). Effects of N-glycosylation on the
biochemical properties of recombinant bEKL expressed in Pichia pastoris. Enzyme Microb. Technol..

[18] Chang X., Xu B., Bai Y., Luo H., Ma R., Shi P., Yao B. (2017). Role of N-linked glycosylation
in the enzymatic properties of a thermophilic
GH 10 xylanase from Aspergillus fumigatus expressed in Pichia pastoris. PLoS One.

[19] Dagar V. K., Babbal, Mohanty S., Khasa Y. P. (2022). Effect of N-glycosylation
on secretion, stability,
and biological activity of recombinant human interleukin-3 (hIL-3)
in Pichia pastoris. 3 Biotech.

[20] Mikolajczyk K., Kaczmarek R., Czerwinski M. (2020). How glycosylation
affects glycosylation:
The role of N-glycans in glycosyltransferase activity. Glycobiology.

[21] Zhang W., Bevins M. A., Plantz B. A., Smith L. A., Meagher M. M. (2000). Modeling
Pichia pastoris growth on methanol and optimizing the production of
a recombinant protein, the heavy-chain fragment C of botulinum neurotoxin,
serotype A. Biotechnol. Bioeng..

[22] Werten M. W. T., Moers A. P. H. A., Vong T. H., Zuilhof H., van Hest J. C. M., de
Wolf F. A. (2008). Biosynthesis of an amphiphilic silk-like
polymer. Biomacromolecules.

[23] Stargardt P., Feuchtenhofer L., Cserjan-Puschmann M., Striedner G., Mairhofer J. (2020). Bacteriophage Inspired Growth-Decoupled Recombinant
Protein Production in Escherichia coli. ACS
Synth. Biol..

[24] DeLano, W. L. PyMOL: An open-source molecular graphics tool. CCP4 Newsl Protein Crystallogr., 2002; Vol. 40, pp 82–92.

[25] Turupcu, A. Molecular Simulations of Glycans and Glycoproteins, Dissertation, Vienna, Austria, 2019.

[26] Turupcu A., Oostenbrink C. (2017). Modeling of
Oligosaccharides within Glycoproteins from
Free-Energy Landscapes. J. Chem. Inf. Model..

[27] Kabsch W. (2010). XDS. Acta Crystallogr.,
Sect. D:Biol. Crystallogr..

[28] Delagenière S. (2011). ISPyB: An information management system
for synchrotron macromolecular
crystallography. Bioinformatics.

[29] Computational C. (1994). The CCP4 Suite:
Programs for Protein Crystallography. Acta Crystallogr..

[30] Vagin A. A., Steiner R. A., Lebedev A. A., Potterton L., McNicholas S., Long F., Murshudov G. N. (2004). REFMAC5
dictionary: Organization of prior chemical knowledge and guidelines
for its use. Acta Crystallogr., Sect. D:Biol.
Crystallogr..

[31] Emsley P., Lohkamp B., Scott W. G., Cowtan K. (2010). Features and development
of Coot. Acta Crystallogr., Sect. D:Biol. Crystallogr..

[32] Liebschner D. (2019). Macromolecular structure
determination using X-rays, neutrons and
electrons: Recent developments in Phenix. Acta
Crystallogr., Sect. D:Struct. Biol..

[33] Radoman B., Grünwald-Gruber C., Schmelzer B., Zavec D., Gasser B., Altmann F., Mattanovich D. (2021). The Degree
and Length of O-Glycosylation of Recombinant Proteins Produced in
Pichia pastoris Depends on the Nature of the Protein and the Process
Type. Biotechnol. J..

[34] Reddy A., Gibbs B. S., Liu Y. L., Coward J. K., Changchien L. M., Maley F. (1999). Glycosylation of the overlapping sequons in yeast external invertase:
effect of amino acid variation on site selectivity in vivo and in
vitro. Glycobiology.

[35] Nishida H., Tokiwa Y. (1993). Effects of Higher-Order Structure
of Poly­(3-hydroxybutyrate)
on Its Biodegradation. II. Effects of Crystal Structure on Microbial
Degradation. J. Environ. Polym. Degrad..

[36] Huang, S. J. 21 Biodegradation. In Comprehensive Polymer Science and Supplements; Elsevier, 1989; Vol. 6, pp 597–606.

[37] Tokiwa Y., Suzuki T. (1981). Hydrolysis of Copolyesters Containing Aromatic and
Aliphatic Ester Blocks by Lipase. J. Appl. Polym.
Sci..

[38] Ronkvist Å. M., Xie W., Lu W., Gross R. A. (2009). Cutinase-Catalyzed
hydrolysis of poly­(ethylene terephthalate). Macromolecules.

[39] Thiyagarajan S., Maaskant-Reilink E., Ewing T. A., Julsing M. K., Van Haveren J. (2021). Back-to-monomer
recycling of polycondensation polymers: Opportunities for chemicals
and enzymes. RSC Adv..

[40] Shental-Bechor D., Levy Y. (2009). Folding of glycoproteins: toward understanding the biophysics of
the glycosylation code. Curr. Opin. Struct.
Biol..

[41] Sun B., Hood L. (2014). Protein-centric N-glycoproteomics
analysis of membrane and plasma
membrane proteins. J. Proteome Res..

[42] Sparbier K., Koch S., Kessler I., Wenzel T., Kostrzewa M. (2005). Selective
Isolation of Glycoproteins and Glycopeptides for MALDI-TOF MS Detection
Supported by Magnetic Particles. J. Biomol.
Tech..

[43] Shirke A. N. (2016). Influence of surface charge, binding site residues and glycosylation
on Thielavia terrestris cutinase biochemical characteristics. Appl. Microbiol. Biotechnol..

[44] Skropeta D. (2009). The effect
of individual N-glycans on enzyme activity. Bioorg. Med. Chem..

[45] Brondyk W. H. (2009). Chapter
11 Selecting an Appropriate Method for Expressing a Recombinant Protein. Methods Enzymol..

[46] Cregg J. M., Tolstorukov I., Kusari A., Sunga J., Madden K., Chappell T. (2009). Chapter 13
Expression in the Yeast Pichia pastoris. Methods
Enzymol..

[47] Pfaff L. (2022). Multiple Substrate Binding
Mode-Guided Engineering of a Thermophilic
PET Hydrolase. ACS Catal..

