# Supplementary material for: Trade-Offs between Stability and Activity of Glycosylated and Non-Glycosylated Polyester Hydrolases PHL7 and PHL7mut3
Source: ACS ES T Eng. 2025 Aug 7;5(11):2781–91. doi: 10.1021/acsestengg.5c00272 (PMC12624734; doi:10.1021/acsestengg.5c00272)
Supplement: Supplementary file 1 [file ee5c00272_si_001.pdf]

Supplementary information

Trade-offs between stability and activity of glycosylated and non-glycosylated polyester hydrolases PHL7 and PHL7mut3

[Lisa Fohler][1], [Felix Faschingeder][1], [Lukas Leibetseder][1], [Ziyue Zhao][2], [Abibe Useini][3], [Norbert Sträter][3], [Christian Sonnendecker][2], [Tom A. Ewing][4], [Antoine P. H. A. Moers] [4], [Marc W. T. Werten] [4], [Daan M. van Vliet][4], [Mattijs K. Julsing][4], [Wolfgang Zimmermann][2], [Gerald Striedner][1] *

[1] [Department of Biotechnology, Institute of Bioprocess Science and Engineering, BOKU University, Vienna, 1190, Austria]

[2] [Institute of Analytical Chemistry, Leipzig University, Leipzig, 04103, Germany]

[3][Institute of Bioanalytical Chemistry, Center for Biotechnology and Biomedicine, Leipzig University, Leipzig, 04103, Germany]

[4] [Wageningen Food & Biobased Research, Wageningen University & Research, Wageningen, 6708, The Netherlands]

Correspondence: [Prof. Gerald Striedner] ([gerald.striedner@boku.ac.at]). [Department of Biotechnology, Institute of Bioprocess Science and Engineering, University of Natural Resources and Life Sciences, Muthgasse 18, 1190 Vienna, Austria].

S 1: Amino acid sequences for all six enzymes used in this study

E_PHL7 (expressed in *E. coli*)

MANPYERGPDPTESSIEAVRGPFAVAQTTVSRLQADGFGGGTIYYPTDTSQGTFGAVAISPGFTAGQESIAWLGP
RIASQGFVVITIDTITRLDQPDSRGRQLQAALDHLRTNSVVRNRIDPNRMAVMGHSMGGGGALSAAAANNTSLEAA
IPLQGWHTRKNWSSVRTPTLVVGAQLDTIAPVSSHSEAFYNSLPSDLDKAYMELRGASHLVSNTPDTTTAKYSIA
WLKRFVDDDLRYEQFLCPAPDDFAISEYRSTCPF**LE**HHHHHH

P_PHL7 (expressed in *P. pastoris*)

ANPYERGPDPTESSIEAVRGPFAVAQTTVSRLQADGFGGGTIYYPTDTSQGTFGAVAISPGFTAGQESIAWLGPR
IASQGFVVITIDTITRLDQPDSRGRQLQAALDHLRTNSVVRNRIDPNRMAVMGHSMGGGGALSAAA**NN**TSLEAAI
PLQGWHTRK**N**WSSVRTPTLVVGAQLDTIAPVSSHSEAFYNSLPSDLDKAYMELRGASHLVSNTPDTTTAKYSIAW
LKRFVDDDLRYEQFLCPAPDDFAISEYRSTCPFHHHHHH

P_PHL7_ng (expressed in *P. pastoris*)

ANPYERGPDPTESSIEAVRGPFAVAQTTVSRLQADGFGGGTIYYPTDTSQGTFGAVAISPGFTAGQESIAWLGPR
IASQGFVVITIDTITRLDQPDSRGRQLQAALDHLRTNSVVRNRIDPNRMAVMGHSMGGGGALSAAA**QQ**TSLEAAI
PLQGWHTRKQWSSVRTPTLVVGAQLDTIAPVSSHSEAFYNSLPSDLDKAYMELRGASHLVSNTPDTTTAKYSIAW
LKRFVDDDLRYEQFLCPAPDDFAISEYRSTCPFHHHHHH

E_PHL7mut3 (expressed in *E. coli*)

MANPYERGPDPTESSIEAVRGPFAVAQTTVSRLQADGFGGGTIYYPTDTSQGTFGAVAISPGFTAGQESIAWLGP
RIASQGFVVITIDTITRLDQPDSRGRQLQAALDHLRTNSVVRNRIDPNRMAVMGHSMGGGGALSAAAANNTSLEAA
IPLQGWHTRKNWSSVRTPTLVVGA**E**LDTIAPVSSHSEAFYNSLPSDLDKAYMELRGASH**T**VSNTPDTTTAKYSIA
WLKRFVD**K**DLRYEQFLCPAPDDFAISEYRSTCPF**LE**HHHHHH

P_PHL7mut3 (expressed in *P. pastoris*)

ANPYERGPDPTESSIEAVRGPFAVAQTTVSRLQADGFGGGTIYYPTDTSQGTFGAVAISPGFTAGQESIAWLGPR
IASQGFVVITIDTITRLDQPDSRGRQLQAALDHLRTNSVVRNRIDPNRMAVMGHSMGGGGALSAAA**NN**TSLEAAI
PLQGWHTRK**N**WSSVRTPTLVVGA**E**LDTIAPVSSHSEAFYNSLPSDLDKAYMELRGASH**T**VSNTPDTTTAKYSIAW
LKRFVD**K**DLRYEQFLCPAPDDFAISEYRSTCPFHHHHHH

P_PHL7mut3_ng (expressed in *P. pastoris*)

ANPYERGPDPTESSIEAVRGPFAVAQTTVSRLQADGFGGGTIYYPTDTSQGTFGAVAISPGFTAGQESIAWLGPR
IASQGFVVITIDTITRLDQPDSRGRQLQAALDHLRTNSVVRNRIDPNRMAVMGHSMGGGGALSAAA**QQ**TSLEAAI
PLQGWHTRK**Q**WSSVRTPTLVVGA**E**LDTIAPVSSHSEAFYNSLPSDLDKAYMELRGASH**T**VSNTPDTTTAKYSIAW
LKRFVD**K**DLRYEQFLCPAPDDFAISEYRSTCPFHHHHHH

Predicted N-glycosylation sites

Observed N-glycosylation sites

Mutations for higher activity and temperature stability

Mutations for non-glycosylated version

Linker to His-Tag in construct used for *E. coli* expression

Enzyme	T_{on} [°C]	T_m [°C]
E_PHL7	75.4	85.5
P_PHL7	73.7	83.7
P_PHL7_ng	71.1	80.9
E_PHL7mut3	76.6	89.1
P_PHL7mut3	84.5	93.3
P_PHL7mut3_ng	76.6	86.9

S2: Onset of denaturation and inflection point of PHL7 and PHL7mut3 produced in E. coli (E) and P. pastoris (P) in 1 M potassium phosphate buffer measured with DSC.

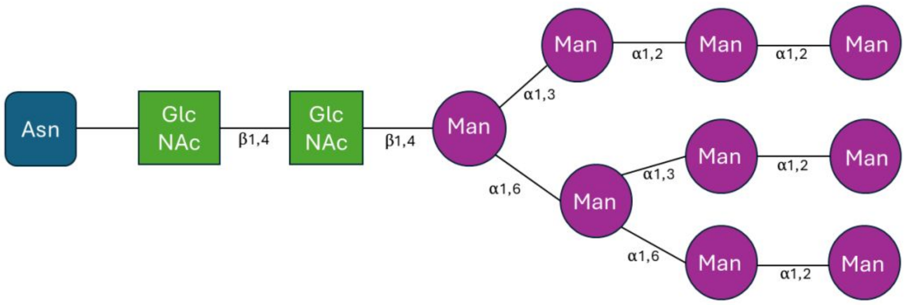

S3: Mannose-9 N-linked oligosaccharide used for modelling of possible conformations linked to PHL7mut3

S4: Diffraction data and refinement statistics

Compound	E_PHL7mut3	P_PHL7mut3	P_PHL7mut3_ng
PDB entry ID	9QDV	9QBN	9QDE
Data collection			
Source	DESY EMBL P13	DESY EMBL P13	DESY EMBL P13
Wavelength (Å)	0.7300	0.9762	0.9762
Resolution (Å)	75.72-0.89 (0.96-0.89)	101.03-1.41 (1.49-1.41)	48.69-1.02 (1.12-1.02)
Resolution aniso (Å)	0.910, 0.894, 1.04	1.432, 1.407, 1.53	1.099, 1.188, 1.03
Space group	C 1 2 1	$P2_12_12_1$	$P2_12_12_1$
Unit cell dimensions	150.44, 43.90, 100.12;	45.42, 64.00, 101.03;	46.05, 59.01, 86.15;

(Å;°)	90.000, 130.867 90.000	90.0, 90.0 90.0	90.0, 90.0 90.0
Unique reflections	292736 (14640)	47787 (2389)	90273 (4515)
Multiplicity	6.9 (7.3)	8.0 (6.6)	12.8 (7.9)
Completeness (%)* spherical/ellipsoidal	78.7 (21.0) / 92.5 (57.5)	82.3 (25.2) / 89.8 (44.7)	76.4 (16.9) / 93.7 (59.1)
Mean I/σ(I)	9.5 (1.5)	7.7 (1.4)	18.4 (1.6)
R-meas	0.098 (1.203)	0.213 (1.492)	0.058 (1.225)
R-merge	0.091 (1.118)	0.200 (1.376)	0.055 (1.147)
R-pim	0.037 (0.444)	0.073 (0.566)	0.016 (0.420)
$CC_{1/2}$	0.997 (0.633)	0.995 (0.375)	0.999 (0.566)
Wilson B (Å^2)	8.53	11.76	11.75
Refinement			
Resolution (Å)	32.93-0.89 (0.90-0.89)	41.43-1.37 (1.40-1.37)	27.92-1.02 (1.05-1.02)
R-work	0.1185 (0.2611)	0.1321 (0.2948)	0.1238 (0.2873)
R-free	0.1311 (0.2602)	0.1690 (0.3251)	0.1496 (0.4236)
Number of non-hydrogen atoms, B-value (Å^2)			
Protein	4201, 10.92	2080, 12.51	2103, 15.13
Heterogen	0, 0.00	38, 24.36	1, 24.57
Solvent	856, 25.89	472, 30.70	458, 31.13
Rmsd bonds (Å), angles (°)	0.010, 1.196	0.004, 0.804	0.011, 1.125
Ramachandran favored, allowed, outliers (%)	97.66, 2.34, 0.00	98.47, 1.53, 0.00	98.84, 1.16, 0.00
Rotamer outliers (%)	0.66	0.0	0.44
MolProbity clashscore	0.60	0.00	0.00

*Anisotropic truncation has been used. The first line refers to the spherical and the second line to the ellipsoidal completeness.

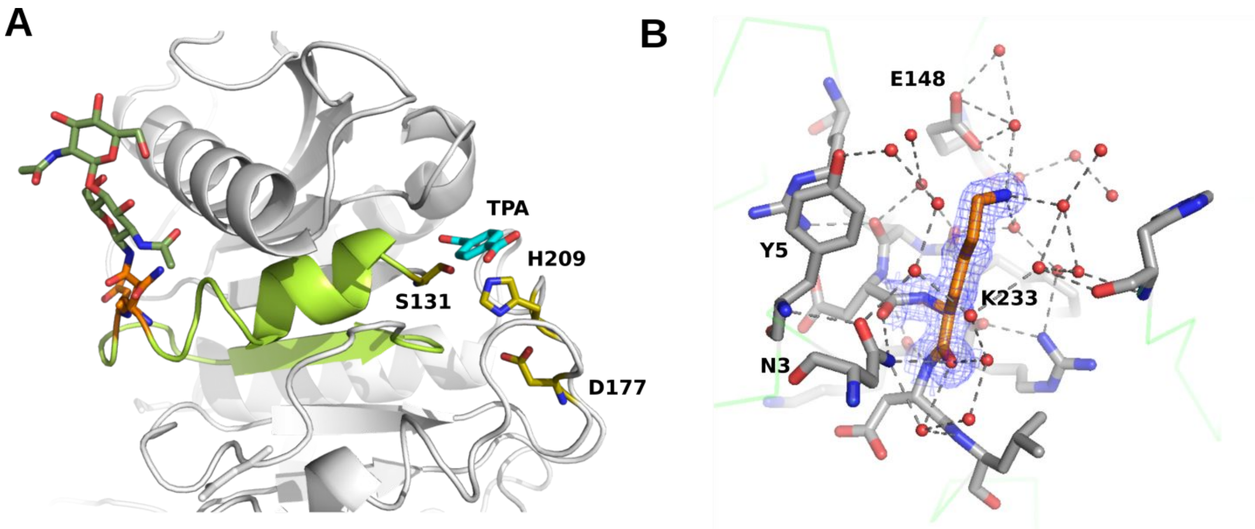

S5: A - Crystal structure of P_PHL7mut3. The glycan is displayed in dark green, N143 in orange and the active site is marked by terephthalic acid (TPA, cyan) and catalytic triad residues (gold). The helix and ß-strand connected by the loop where the glycan binds is presented in green. TPA has been superimposed from the PHL7×TPA co-crystal structure (pdb id 8bra). B - Interaction of K233 with nearby residues and solvent. Water molecules are displayed as red spheres.
